# Attracting and retaining physicians in less attractive specialties: the role of continuing medical education

**DOI:** 10.1186/s12960-021-00613-z

**Published:** 2021-05-19

**Authors:** Van Anh Thi Nguyen, Karen D. Könings, Albert J. J. A. Scherpbier, Jeroen J. G. van Merriënboer

**Affiliations:** 1 Department of Medical Education and Skills Laboratory, Hanoi Medical University, Room 504, B Building, 1 Ton That Tung Street, Dongda, Hanoi, 10000 Vietnam; 2 School of Health Professions Education, Faculty of Health, Medicine and Life Sciences, Maastricht University, P.O. Box 616, 6200 MD Maastricht, The Netherlands

**Keywords:** Guidelines, Continuing medical education, CME, Attraction, Retention, Physicians, Less attractive specialties

## Abstract

**Background:**

Less attractive specialties in medicine are struggling to recruit and retain physicians. When properly organized and delivered, continuing medical education (CME) activities that include short courses, coaching in the workplace, and communities of practice might offer a solution to this problem. This position paper discusses how educationalists can create CME activities based on the self-determination theory that increase physicians’ intrinsic motivation to work in these specialties.

**Main content:**

The authors propose a set of guidelines for the design of CME activities that offer physicians meaningful training experiences within the limits of the available resources and support. First, to increase physicians’ sense of professional relatedness, educationalists must conduct a learner needs assessment, evaluate CME’s long-term outcomes in work-based settings, create social learning networks, and involve stakeholders in every step of the CME design and implementation process. Moreover, providing accessible, practical training formats and giving informative performance feedback that authentically connects to learners' working life situation increases physicians’ competence and autonomy, so that they can confidently and independently manage the situations in their practice contexts. For each guideline, application methods and instruments are proposed, making use of relevant literature and connecting to the self-determination theory.

**Conclusions:**

By reducing feelings of professional isolation and reinforcing feelings of competence and autonomy in physicians, CME activities show promise as a strategy to recruit and retain physicians in less attractive specialties.

## Introduction

Imbalance in the health workforce is no novelty; it is reported in most of the health care professions in both developed and developing countries [1]. Previous studies have reported imbalances between specialties (generalists vs. specialists), services (preventive vs. curative care), gender, and geographical location (urban vs. rural areas) [1, 2]. In fact, shortages of physicians have been reported mainly in primary care specialties and in specialties that provide preventive care services in the community [3–5]. In the United States, 88% of doctors are specialists, while only 12% of doctors are generalists (i.e., general practitioners, family doctors, and other non-specialist medical practitioners). Similar percentages of 72–28% have been reported in the United Kingdom [4]. This maldistribution is even more pronounced in developing countries, where the demand for professionals in many important primary health care occupations far exceeds their supply [5–7]. For instance, to meet the mental health care needs of the African and South-East Asian populations, at least 20 additional mental health professionals are required per 100,000 inhabitants, and most of the psychiatrists work for mental hospitals [5]. Or in case of family medicine, despite its important role in low- and middle-income countries (LMICs)’ health system to achieve health equity and attain Sustainable Development Goals [8], it is still relatively new and has not been a residency-based medical specialty in many African and Asian countries [9]. In other areas, such as general practitioner, geriatrics, pediatrics, and preventive medicine, similar maldistributions of physicians are recorded [10–13]. Several reasons are listed to explain for the preponderance of specialist over generalist or "preventist" doctors, such as: less medical students choose non-specialist specialties [11, 14], low job satisfaction of generalist doctors due to low incomes [6, 7, 15], high work load [7, 12, 16], as well as perceiving low prestige compared to specialist doctors [16, 17].

As a consequence of this imbalance, physicians who do work in less attractive specialties (LASs) face a higher workload, feelings of professional isolation, limited career development opportunities, and economic instability [18]. These unfavorable conditions, in turn, make physicians less satisfied and willing to remain in LASs [19], creating a vicious cycle of health care workforce imbalance and poor health outcomes of the population, especially in the primary care domains [16, 20, 21]. Indeed, physicians working in LASs are extremely disappointed with their salary which is significantly lower than that of their colleagues in other specialties [2, 22, 23]. Even in the LMICs, where all doctors are paid a flat rate regardless of specialty, such as Nigeria [24] or Vietnam [7], physicians working in LASs (such as primary care, mental health or prentive medicine) do not satisfy with their incomes. They do not have opportunities to get paid from other allowances (i.e., private practice, perdiem for attending workshop, etc.) as much as their clinical colleagues do [7, 16, 24]. Although financial incentives have had a positive impact on the recruitment of doctors [25, 26], as extrinsic motivators, they did not have a long-lasting effect [27, 28]. LASs physicians typically work in challenging environments [12] with limited resources or infrastructures [29] and a lack of supervision and connection to their professional community [30]. Although efforts to improve health care infrastructure and physicians’ working and living conditions have been found to enhance job satisfaction and retention [30], their effectiveness is limited as they require considerable skills in managing and monitoring the allocation and use of resources, especially in developing countries [31]. Other interventions to provide personal and professional support in the form of close mentoring and supervision have had positive results in increasing primary care physicians’ performance and job satisfaction [31–33]. Yet, we need more follow-up studies to evaluate the impact on physicians’ intention to continue working in the field [33].

Another demotivating factor is that physicians in LASs have fewer opportunities to participate in continuing medical education (CME) activities, such as classes, seminars, and training, to update their knowledge and extend skills in their field. Indeed, CME is considered an important factor motivating doctors to work in LASs [7, 10, 34], as it significantly improves satisfaction, learning, performance, and specialist recertification in LASs physicians [30–33]. For example, physicians working in rural Kenya and Benin feel more comfortable and confident with their work taking short training courses, with about 20% of them mentioning an increase of interest and work commitment [31]. This effectiveness of CME on satisfaction and retention of physicians working in LASs is also reported in other countries [34–39]. Considering that training programs for physicians in LASs, including in-service training or continuing education, are often not available [40–43], there is a need for information on how CME can be designed and implemented to increase physicians’ motivation to work in LASs.

Recently, most research on the effectiveness of CME for motivating healthcare workers took the form of experimental studies [30, 37, 39], evaluating the impacts on participants’ knowledge and practice as well as patients’ outcomes. CME also was mentioned in several systematic reviews of strategies to cope with the problem of healthcare worker shortages [35, 43]. These studies and reviews provided evidence for the necessity and the effectiveness of CME. However, to highlight the characteristics of CME as a potential solution for recruiting and retaining healthcare workers in LASs, we seek to offer a practical set of guidelines for the design and organization of CME activities in the format of a position paper. Based on Self-Determination Theory (SDT) [44], we purposefully searched the literature for research findings that support our claims and proposed guidelines. First, we will describe how SDT, and, more specifically, its three components of Autonomy, Competence, and Relatedness representing physicians’ psychological needs that must be addressed, can be applied to the design and implementation of CME activities for LASs. We will then present the guidelines for the said CME activities, providing specific directions for each of the following three stages of development: (1) goal setting and evaluation, (2) design and development, and (3) implementation of the learning activities involving all relevant stakeholders. Finally, in the Discussion section we will present specific practical tips and set out the implications of these guidelines.

## Self-determination theory

**Fig. 1:**
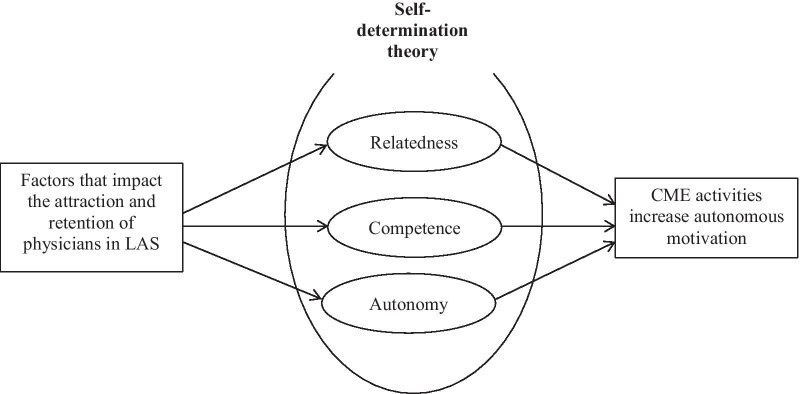
Framework for developing CME activities based on the self-determination theory that effectively motivate physicians to work and remain in LASs

Presented by Deci and Ryan in 1985, SDT has been widely used as a theory of work motivation in various contexts, such as business, education, and health care, and has recently been introduced into the medical education discourse as well [45, 46]. It differs from other theories of motivation in that it distinguishes a spectrum of motivation ranging from amotivation (total disengagement), through extrinsic motivation or controlled motivation (people undertake an activity under pressure, often expecting extrinsic rewards to stay motivated), to intrinsic motivation or autonomous motivation (people engage in an activity, because they find it interesting). According to SDT, to foster motivation the following three psychological needs of an individual must be addressed: *autonomy* (the feeling of being free to choose whatever one desires); *competence* (the feeling of being effective in whatever action one performs), and *relatedness* (the feeling of being connected with or belonging to and accepted by one’s community) [44]. As such, SDT presents a comprehensive framework for analyzing how effective CME activities can help cultivate the autonomous motivation in LASs physicians to enter and stay active in the fields by increasing these feelings of relatedness, competence, and autonomy (see Fig. 1).

Physicians in LASs usually have to work independently, isolated from their professional community [47–49]. Such professional communities are generally sustained by means of informal gatherings of physicians in their workplace or professional organizations; they are also formally formed via hierarchical work relations or by taking part in conference meetings or CME activities. A study with Canadian family doctors revealed that feeling related was the largest contributor to physicians’ job satisfaction and work-related engagement [49]. The working conditions in LASs are such, however, that physicians have less professional connections, thereby reducing their opportunities to talk with colleagues about their work, their patients, and to seek their support, empathy, or simply the passion and motivation to work [48]. Moreover, working conditions in LASs usually require a high sense of self-confidence and independence in physicians’ performance, which underscores their autonomy and competence needs. Indeed, an international study involving primary care physicians in the USA, UK, and Germany found that a lack of administrative and clinical autonomy was associated with high levels of work stress in physicians in all three health care systems [50].

It has been suggested that to increase physicians’ feelings of relatedness, it is important to first carefully analyze their learning needs [51] and to connect them with their community [49, 52]. To strengthen their feelings of competence, moreover, physicians should be continuously updated with new knowledge and practice [53] and receive close supervision via on-site follow-up and evaluation [54]. Finally, to enhance feelings of autonomy, physicians must be free to study in their own setting, undertake learning activities that are learner-centered and practical [35], and have the opportunity to independently apply new knowledge and skills to their own struggles in daily practice [37, 55]. In sum, these three basic psychological needs of physicians—relatedness, competence, and autonomy—should be considered and embedded in every step of the CME design and implementation process to increase autonomous motivation in LASs physicians and induce them to consistently pursue their careers in LASs.

## CME guidelines

In the following section, we propose a set of SDT-inspired guidelines for the design of CME activities that help attract physicians to LASs and retain them. The relevant literature used to support these guidelines was searched for in PubMed, Web of Sciences, and Google Scholar. Table 1 gives an overview of these specific guidelines, which are organized into three parts. While the first part focuses on how to define and evaluate the objectives of CME, the second part targets the design and development of learning activities that address these goals. Finally, the third part explains how to involve all relevant stakeholders in the implementation process.

**Table 1:** CME guidelines that may help to recruit and retain health professionals in LASs

Scope	Guidelines	Alignment with Self-Determination Theory
Goal setting and evaluation	– Keep physicians up-to-date with current and best practices in their specialty by addressing their specific needs – Conduct a needs assessment and evaluation both online and offline, via alumni networks or professional associations	Increase relatedness
	– Use evaluation methods that authentically connect to learners' working life situation to stimulate learners to appreciate the effectiveness of their new learning and to build their capacities, so that they can confidently and independently manage the situations in their practice context	Increase competence and autonomy
Design and development of learning activities	– Use formats that are learner-centered which will increase participants’ independence, provided they can practice and apply new knowledge and skills in their own situation and also learn from their own struggles in daily practice – Train LASs physicians in practical, flexible, and user-friendly platforms, so that they can adapt learning to their busy, isolated, and resource-limited settings	Increase autonomy and competence
	– Give doctors equal and fair opportunities to participate in CME, regardless of their position and experience; Let participants share the knowledge acquired with colleagues in their organization – Create a social learning network and community of practice among participants during and after the educational activities	Increase relatedness
Involvement of stakeholders in the implementation process	– Develop alumni networks and professional associations and engage these in every step of the CME development process by making use of their contributions and following their interests	Increase relatedness
	– Involve facilitators from health professions education institutions and organizations and technologists in the development of CME – Develop a policy of licensing and continuing professional development requirements, including a quality assurance procedure for designing CME courses	Increase competence

## Goal setting and evaluation

As can be seen in Table 1, to be able to develop CME activities that enhance feelings of relatedness in LASs physicians, it is important that participants’ training needs be carefully analyzed via their specialty’s professional community and alumni networks, both online and offline. Similarly, feelings of competence and autonomy can be reinforced in LASs physicians, by making sure the design of CME activities includes an evaluation of their impact on physicians’ practice, attitudes, and behaviors in their daily practice as well as on their connection and commitment to stay in the specialty. We will now elaborate on each of these aspects.

### Analyze doctors' needs

In conducting a needs assessment, it is important to differentiate between doctors’ “felt,” “perceived,” and “expressed” learning needs. Felt needs are what doctors feel they need, based on their own experiences in direct patient care. Perceived needs refer to what they take in after having interacted with colleagues and the professional community in clinical and academic activities. Finally, expressed needs are what they report in a formal needs analysis conducted by their organization for quality management and risk assessment purposes [56]. Indeed, the literature has shown that doctors working in LASs have various learning needs, which range from the need to learn basic medical topics to learning more personal and professional competencies for high-quality practice. Primary care doctors in developing countries, for instance, need to learn basic knowledge of how to diagnose and manage common diseases in the community [57]. Their colleagues in developed countries, on the other hand, need to learn about disease pattern changes or other additional knowledge to manage patients in new emerging fields in their area, as in the case of Canadian family doctors wanting to learn genetic counseling [53] or listeriosis care during pregnancy [58]. Furthermore, LASs physicians in developed countries also want to learn about teaching and teamwork [59] or how to provide palliative care for patients in rural and remote locations, where many people prefer care at home during their end-of-life phase [37]. When based on instructional approaches that address these specific needs, CME can help to close any existing gaps between doctors’ current and best practices [51].

Depending on LASs physicians’ working conditions, such needs analysis should be conducted either online (e.g., when physicians work in remote areas or are difficult to reach through other channels) or offline (e.g., when physicians cannot access the Internet or can only be reached via professional activities in their specialty). To obtain doctors’ valid contact information, one might refer to the alumni mailing list, a result of previous educational activities, or the professional associations. Using these same alumni networks or professional associations for evaluation purposes, moreover, might reinforce the connections between the organizations and their members, thereby reducing feelings of professional isolation in LASs physicians [37].

### Evaluate the quality of CME activities

It is essential to evaluate whether the CME program has been successful in teaching particular competencies to LASs physicians and in increasing their independent performance at work. Using evaluation formats that “authentically connect to learners’ life circumstances, frame of preference and values”, learners can receive informative feedback on their new competencies, so that they can independently manage situations in their practice context [60]. Case scenarios offer an authentic way to evaluate how learners apply the relevant knowledge learned in the CME courses to similar situations in their work settings [61]. Another evaluation method that is precise and authentic is “Audit and Feedback.” Often used for quality improvement purposes in the workplace, this intervention first measures physicians’ performance and consequently provides them with feedback from instructors, supervisors, or colleagues on specific points needing improvement, accompanied by an appropriate action plan [62]. Over the long term, these workplace-based evaluation methods have the potential to strengthen feelings of relatedness [61], competence, and independence in physicians, which can be considered a true benefit [54, 63]. These long-term effects on physicians’ professional development and careers are often missed by current CME evaluation practices as they mainly focus on outcomes that are easy to measure, such as participants’ self-reported knowledge, confidence, skills, and attitudes [54].

To measure other long-term outcomes of CME, such as learners’ performance, outcomes on patient’s health or community health, Moore et al. [63] proposed a framework for outcomes assessment in CME. This seven-level framework, which was expanded on the base of integrating his original framework and other models of assesment and evaluation, such as Kirkpatrick’s model of training evaluation [64] and Miller’s pyramid [65], can be used to design an assessment plan at each stage of the CME development process. Evaluations of CME programs for LASs physicians should, therefore, be long enough to allow these less tangible outcomes to materialize, especially those that are harder to measure, such as professional growth, networking, or the commitment to stay in the field [54]. Furthermore, the content and formats of CME normally have to be modified to meet the demands of the local situation and specific learners’ needs. For example, a CME course in Advanced Trauma Life Support in developed countries has been changed to the Primary Trauma Care training in LMICs, which have limited resources and different patterns of injury and trauma care workforce [66]. The evaluation of CME activities, therefore, should not only focus on the learning and teaching process and its usefullness outcomes, but also on determining the extend to which objectives of the program are attained while considering the variety of learner’s needs, capacity of educational institutions and available resources to capture a wide variety of effects [67].

## Designing and developing learner-centered activities

To increase participants’ autonomy and competence, CME activities should be offered in learner-centered, practical, and flexible modalities which allow participants to practice and apply new knowledge and skills in their own situation and adapt learning to their busy and isolated working conditions. To increase feelings of relatedness, moreover, CME should create a learning community, where opportunities to study are open and fair to all LASs doctors, independent of their location and position.

### Use formats that are learner-centered and practical

To ensure that CME activities enhance doctors’ competence which translates to improved clinical performance, it is imperative that CME contents be tailored to their individual needs. In addition, the learning activities must be interactive and allow doctors to apply the newly acquired knowledge in their daily practice [43]. This high relevance of the CME study contents and evaluation methods, including their practicality and accessibility, might induce participants to develop a “positive attitude toward learning”, meaning that they become interested or motivated [60]. When focused on the learner, CME formats might also help to foster participants’ self-regulation or autonomy, provided they have the opportunity to practice and apply the knowledge and skills learned in their own situation and to their own struggles in daily practice*.*

The recent use of advanced teaching methods in medical education, accelerated by the rapid development of information and communication technology, has greatly helped to reduce the geographical and professional isolation of LASs physicians. E-learning and Internet-delivered CME activities (e.g., Massive Online Open Courses and Webinars) have brought along several advantages that suit LASs physicians’ working life. For instance, they are convenient, give access to remote areas, are adaptable to doctors’ busy schedules [68, 69], and provide diverse and abundant digital resources [70–72]. As such, these innovative educational technologies have been proved acceptable and effective in delivering physicians knowledge electronically [70, 72]. Likewise, software applications on mobile phones and portable electronic devices (mHealth-mobile Health, mCME-mobile CME, and gaming) have been used as a tool to disseminate information, offer clinical decision support [73, 74], master skills [75] and, combined with feedback in coaching groups, to increase reflection in clinical practice [76]. It should be borne in mind, however, that several factors might impact the application of e-learning or mHealth in CME for LASs physicians, such as a limited scope of training, rapid changes to the applications [77], and lagging human and infrastructural resources which are quite common in remote areas and in developing countries [70, 78].

### Create and maintain a learning community

Providing professional support in the form of relevant educational activities is an effective strategy to reduce isolation and increase retention among LASs doctors [79]. CME is more likely to be valued if it allows for the creation of a social learning network among participants during and after the courses. Using small-group learning in CME, moreover, will afford LASs physicians the opportunity to meet with colleagues from their field and to integrate personal, social, and professional experiences into the learning process [70, 80]. More than 90% of Australian general practitioners reported a preference for learning in a group over self-educating online as it enhanced their feelings of professional relatedness [81]. Also the application of teleconferencing allows physicians, especially those in rural areas, to consult with their colleagues and supervisors online from a distance. By saving time and costs of travel, such approaches effectively address the challenges LASs doctors face, such as personal isolation and a lack of supervision [43].

By staying connected to other alumni of their professional associations or of previous CME activities, physicians in LASs can create their own learning communities. In such communities, physicians who work in big cities could, for instance, teach their peers who cannot easily access the training or they can simply share their past CME experiences with them. Due to ineffective resource allocation and top-down management, junior or practicing LASs doctors, especially those in developing countries, have fewer opportunities to participate in CME, as these activities are often the preserve of managers or senior doctors [82]. Therefore, the idea of creating a community of practice via CME activities, where participants can share the knowledge acquired and train their colleagues could also be applied as a faculty development method [83]. Similarly, such communities of practice could help generate “best” practices to solve common clinical problems [84] and facilitate the implementation of new practices in individual working conditions [85].

## Involving stakeholders in the implementation

As presented in Table 1, it is imperative to involve multiple stakeholders in the whole cycle of CME creation and sustainment to ensure its effectiveness and to autonomously motivate LASs physicians to participate in the activities. Stakeholders should include not only alumni networks, professional associations, educational institutions, and information technology supporters, but also national governments and international organizations.

### Motivate LASs physicians to participate in CME activities

It is crucial to establish alumni networks or professional associations of LASs physicians and to engage and involve them as key informants in all activities related to the design and implementation of CME courses. Preferably, alumni themselves should drive these initiatives and share their learning needs [86]. Physicians’ needs for CME and professional development could be determined by conducting surveys and tracking alumni or physicians’ career paths [87]. Their reflection and feedback will be vital for CME quality assurance and development [88].

In addition, keeping the alumni or professional association members up-to-date and connected to their fields and community will promote their self-determination and motivation to pursue the same professional development goals as their colleagues have [60]. CME could foster feelings of confidence and autonomy in LASs physicians by allowing them to apply new knowledge and skills to their own problems in the workplace [84] or by offering distance supervision and support from more experienced colleagues [43]. Finally, making CME attendance a requirement of the licensing procedure could be a strong extrinsic motivator for LASs physicians [88, 89]. However, to promote learners’ participation in CME activities and boost their personal and professional development, there must be an adequate balance between pressure and support in the work setting [90].

### Involve other stakeholders to keep CME effective

The CME designers should involve and listen to the voice of medical students, doctors yet to enter training and those in training as they are the main beneficiaries as well as important stakeholders of the programs. On the one hand, involving learners in every stage of the teaching and learning process will stimulate their feelings of relatedness [60], ownership and empowerment [91]. On the other hand, the feedback of learners can support formulation of plans for change and improve the quality of education and professional development of teachers [92, 93]. The CME program developers and teachers should support and motivate learners’ involvement in co-creation of education which will bring benefits to all relevant stakeholders [94].

In addition, involving other stakeholders, such as governments, academia, and technologists, as partners in every step of the CME design and development process is indispensable [73, 94]. The government plays a key role in ensuring educational grants and making sure that financial incentives are well allocated and effectively used [82]. It is also instrumental in developing policies to coordinate doctor replacements, freeing study time for physicians [43], and in incorporating CME requirements into licensing/relicensing procedures and continuing professional development of LASs physicians [36, 89]. Medical schools or educational institutions, in their turn, must ensure that CME contents and activities are customized to the needs of the physicians in their regions, and that CME is encouraged and facilitated among LASs physicians via the continuum of subsequent education and training [95]. Not only must these educational institutions equip CME trainers with appropriate teaching skills, they should also teach them community facilitation skills to establish and reinforce professional relatedness among groups of LASs physicians [85]. Although online distance CME has the potential to reduce professional isolation among physicians in LASs and remote areas, its effectiveness depends on the computer skills of facilitators and learners, its accessibility and their acceptance, and on the technological maintenance and user support system [72, 77]. Moreover, when running online CME activities, many other technical issues must be considered, such as security, confidentiality and copyright protection concerns [96]. This is why the involvement of educational technologists in the development, delivery, and implementation of online learning is essential: They can help optimize the uptake of this advanced learning approach in a specific local context [71, 73].

## Discussion

Access to CME is a key factor in attracting physicians to LASs and retaining them. In this article, we proposed guidelines for organizing and implementing CME activities in such a way that they can help to increase physicians’ autonomous motivation to enter and stay active in LASs. By carefully analyzing physicians’ learning needs, and establishing and maintaining a learning community among participants, CME activities could help foster professional relatedness. Whether activities are designed online or in small groups or meetings in the workplace, CME modalities must be flexible, user-friendly, practical, and applicable in LASs physicians’ setting and environment to enhance their competence and autonomy. The involvement of all relevant stakeholders in the design and implementation of CME is vital to engage LASs physicians in CME and to ensure effectiveness of the respective activities in “anchoring” their career commitment.

Although CME has been proved effective as an approach to improve LASs physicians’ satisfaction, learning, and performance [35–37], we recommend that future studies focus on evaluating the long-term impact of CME interventions on LASs physicians’ personal, professional, and career development [54]. Since the problem of recruiting and retaining LASs physicians has been reported globally, in both developed [4, 10] and developing countries [5, 6], the validity and feasibility of the proposed guidelines in either resource-limited or well-established contexts should be tested. In developing countries, for instance, human and infrastructural hurdles could limit the potential of online education [70, 78], whereas a culture of top-down management might inhibit physicians’ freedom to participate in CME activities [82]. Another obstacle is that, although non-specialist physicians recognize the need to update their knowledge and improve their practice through CME, they disagree with the idea of making these CME programs a compulsory part of license renewal procedures in developed countries [90].

In the present paper, we used SDT as a theoretical framework for developing CME guidelines to support LASs physicians’ autonomous motivation. However, SDT cannot account for all aspects contained in the guidelines. Besides offering LASs physicians opportunities to study and practice in their own settings [35, 37, 60], there might be other things we could do to enhance feelings of autonomy in CME participants. More specifically, how can CME continuously nurture and foster LASs physicians’ autonomous motivation? Similar to SDT, we found that Wlodkowski’s “Motivational Framework for Culturally Responsive Teaching” [60] and Keller’s ARCS model of Instructional Design [96] both mention conditions for developing intrinsic motivation. Compared to SDT, however, their approaches are more specific in that they describe how to improve the motivational appeal of instructions (Keller’s ARCS model) or the learning environment per se (Wlodkowski’s framework). These practical strategies and materials could be embedded in the current guidelines with a view to helping CME instructors or designers enhance and maintain the motivation of given target learners, that is, LASs physicians. For example, Wlodkowski’s suggestion to establish *inclusion* in CME activities could be applied by letting instructors and learners, who are often both LASs physicians, collaborate and share own experiences in solving specific problems, thereby creating mutual respect and two-way connections. Keller’s ARCS model, on the other hand, presents several strategies to improve learner *satisfaction* which could make LASs physicians feel good about their accomplishments in CME activities, such as giving appropriate extrinsic rewards without overcontrolling. Such strategies could prevent resentment and reduced enjoyment of the learning activities by physicians who only participate in CME activities to meet the administrative requirements [97].

Informed by SDT, the current guidelines provide a practical basis for developing CME activities that address LASs physicians’ psychological needs of feeling related to the professional community, feeling competent to properly do their work, and feeling autonomous in shaping this work. Other alternative motivational or adult learning theories might be helpful to further refine these guidelines and make them better applicable as a solution to attract more physicians and retain them as active workers in less attractive medical fields.

## Data Availability

Not applicable.

## Abbreviations

LASs: Less attractive specialties
CME: Continuing medical education
SDT: Self-determination theory
